# PotD contributes to *Streptococcus suis*-induced blood–brain barrier disruption by regulating *arcA* transcription

**DOI:** 10.1186/s13567-025-01676-9

**Published:** 2025-12-01

**Authors:** Shiqi Lang, Hang Yin, Xiaoyu Jia, Xiaoying Yu, Anqi Meng, Ru Yan, Juan Li, Lianci Peng, Rendong Fang

**Affiliations:** Joint International Research Laboratory of Animal Health and Animal Food Safety, College of Veterinary Medicine, Southwest University, No.2 Tiansheng Road, Beibei District, Chongqing, 400715 China

**Keywords:** *Streptococcus suis*, PotD, arginine deiminase (ADI), blood–brain barrier (BBB)

## Abstract

The ABC transporter substrate-binding protein PotD plays an important role in bacteria for polyamine uptake, but its role in the pathogenicity of *Streptococcus suis* (*S. suis*) in the host is still unknown. Our study investigated the mechanism by which PotD mediates *S. suis* pathogenicity, especially *S. suis*-induced blood–brain barrier (BBB) disruption. The results showed that Δ*potD* mutant significantly reduced biofilm formation and bacterial load in different tissues, including brain, blood, liver, and lung. Importantly, knockout of *potD* significantly improved mouse survival rate, indicating that PotD is involved in *S. suis* pathogenicity. The deletion of *potD* significantly reversed the reduction of tight junction proteins ZO-1 and Occludin in mouse brain and human cerebral microvascular endothelial cell line D3 (hCMEC/D3), and attenuated BBB disruption via Evans blue (EB) staining assay. Notably, recombinant PotD protein exhibited the disruption of BBB by downregulating ZO-1 and Occludin in hCMEC/D3 cells, demonstrating that PotD contributes to *S. suis*-induced BBB disruption. Furthermore, mass spectrometry analysis and pulldown assay revealed that PotD interacted with arginine deiminase (ADI) encoded by *arcA* and mediated *arcA* transcription. In addition, Δ*arcA* mutant attenuated BBB disruption, and recombinant ADI induced BBB disruption by downregulating ZO-1 and Occludin in hCMEC/D3 cells. Our study reveals the destructive role of the virulence factors *potD* and *arcA* of *S. suis* on the BBB and provides new insights into *S. suis* pathogenicity.

**Supplementary Information:**

The online version contains supplementary material available at 10.1186/s13567-025-01676-9.

## Introduction

*Streptococcus suis* (*S. suis*) is an important zoonotic pathogen that poses a health threat to both humans and pigs [1]. *S. suis* causes sepsis, meningitis, arthritis, and endocarditis in pigs. It can be transmitted to humans through contact with infected animals or contaminated raw pork products, causing meningitis, infectious shock, and even death [2]. Among these pathogenic phenotypes, meningitis is one of the severest phenotypes. Many virulence factors of *S. suis*, such as capsular polysaccharide (CPS), suilysin (Sly), and muramidase-released protein (MRP), have been identified [3]. Some virulence factors facilitate brain endothelial cell adhesion and disrupt the blood–brain barrier (BBB), which triggers the release of inflammatory factors and leads to neuroinflammation [4]. Targeting virulence is an effective strategy for the development of new drugs to combat its infection. Determining the mechanism of its pathogenesis in depth through virulence factors identification would contribute to the discovery of new anti-infective agents.

Polyamines such as spermidine (Spd), spermine (Spm), and putrescine (Put), a class of polycationic organic molecules, are required for optimal growth of bacteria [5]. In bacteria, exogenous polyamines are transported by a well-conserved ATP-binding cassette (ABC) transport system PotABCD, wherein PotD serves as the direct binding receptor for uptake of polyamines [6]. In addition to transporting preferred polyamines, PotD has been identified as a virulence-related protein, which mediates bacterial pathogenicity by different mechanisms. Deletion of *potD* reduces oxidative stress tolerance and biofilm formation in *Streptococcus pneumoniae* (*S. pneumoniae*) [7, 8]. Overexpression of PotD promotes biofilm formation in *Escherichia coli* (*E. coli*) [9]. Deletion of *potD* in *Legionella pneumophila* (*L. pneumophila*) results in sodium (Na^+^) hypersensitivity and impaired infectivity [10]. In *S. suis*, PotABCD has been identified to be responsible for the uptake of polyamines, and PotD binds to the protein of polyamines. In addition, the gene *murB* co-transcribed with PotABCD, mediates peptidoglycan (PG) synthesis of *S. suis*, and deletion of the *potA* gene causes abnormal cell division morphology [11]. However, the role of PotD and its regulatory mechanism on *S. suis* pathogenicity still remain unknown.

Arginine deiminase system (ADS), a secondary metabolic pathway, has been known to be involved in bacterial infection, conferring bacterial acid resistance. ADS degrades arginine into citrulline, ornithine, ammonia, carbon dioxide, and ATP, wherein arginine deiminase (ADI) is the key enzyme converting arginine into citrulline. The conversion of arginine to ornithine generates ATP for bacterial survival in nutrient starvation [12]. ADS in *S. suis* has been identified to be encoded by the *arcABC* operon, in which ArgR is an essential transcriptional regulator of ADS to interact with the *arcABC* operon for bacterial survival in acidic environment [13]. However, whether PotD mediates ADI and PotD-mediated ADI regulates *S. suis* pathogenicity is still unclear.

Therefore, in this study, we constructed *potD* and *arcA* knockout strains (Δ*potD* and Δ*arcA*) to investigate the role of *potD* and *arcA* in *S. suis* pathogenicity. We found that PotD aggravates *S. suis*-induced BBB disruption through regulating *arcA* transcription. Our study provides new insights into PotD-mediated bacterial pathogenicity.

## Materials and methods

### Ethic statements

Female wild-type (WT) C57BL/6 mice were purchased from Chongqing Byrness Weil Biotech Ltd. All the mice were maintained under specific pathogen-free (SPF) conditions before being used at 8–10 weeks of age. All the animal experiments were approved by the Southwest University Ethics Committee, Chongqing, China (IACUC-20231215-02).

### Construction of plasmids

The constructed plasmids included pSET4s-potD, pSET2-potD, pET-28a-potD, and pGEX-4 T-arcA. The *potD* gene from *S. suis* SC19 was cloned into pSET4s and pSET2 by homologous recombination. The *potD* and *arcA* genes from SC19 were cloned into pET-28a (for His-tagged fusion) and pGEX-4 T-1 (for glutathione *S*-transferase [GST]-tagged fusion), respectively. All constructs were confirmed by DNA sequencing. All primers used for the construction of plasmids are shown in Additional file 1. These mutants, constructed using standard molecular biology techniques, were stored in our laboratory.

### Bacterial strains, cell line, and culture conditions

The *S. suis* serotype 2 strain SC19 (GenBank Assembly accession no. GCA_002111405.1) was provided by Prof. Xiangru Wang from Huazhong Agricultural University (Wuhan, China) [14]. The *potD *and *arcA* knockout mutants (Δ*potD* and Δ*arcA*) and their complemented strains (CΔ*potD* and *C*Δ*arcA*) were constructed on the SC19 background in this study (see below for construction details). The prevalent strain of *S. suis* SC19 used in this study was cultured in Todd–Hewitt broth (THB; OXOID, Basingstoke, UK) supplemented with 10% fetal bovine serum (FBS; Gibco, NY, USA) at 37 °C. *E. coli* was cultured in Luria–Bertani broth (LB; HopeBio, Qingdao, China) or LB supplemented with 1.5% agar. To maintain plasmid selection, kanamycin (50 μg/mL; BBI, Shanghai, China) was added to media for the growth of *E. coli* harboring pET-28a; ampicillin (50 μg/mL; BBI, Shanghai, China) was added to media for the growth of *E. coli* harboring pGEX-4 T-1; or spectinomycin (100 μg/mL; BBI, Shanghai, China) was used for the growth of *S. suis* harboring pSET4s and pSET2.

Human cerebral microvascular endothelial cells (hCMEC/D3, Jennio Biotech, Guangzhou, China), mouse brain endothelial cells (bEnd.3; Qingqi Bio, Shanghai, China), and porcine brain microvascular endothelial cells (pBMECs; Procell, Wuhan, China) were cultured at 37 °C with 5% CO_2_. Specifically, hCMEC/D3 and bEnd.3 cells were maintained in Dulbecco’s modified Eagle medium (DMEM; Gibco) supplemented with 10% FBS and 1% penicillin–streptomycin (Gibco). pBMECs were maintained in Porcine Brain Microvascular Endothelial Cell Complete Medium (Procell).

### Construction of *S. suis* mutant strains

The mutant strains, including Δ*potD*, Δ*arcA*, CΔ*potD*, and CΔ*arcA*, were constructed using homologous recombination methods [15, 16]. The *potD* and *arcA* knockout mutants were generated in the SC19 background using the shuttle vector pSET4s. Briefly, the upstream and downstream flanking regions of target genes were amplified by polymerase chain reaction (PCR) using specific primer pairs (potD-1-F/potD-1-R and potD-2-F/potD-2-R for *potD*; arcA-1-F/arcA-1-R and arcA-2-F/arcA-2-R for *arcA*) (Additional file 1). Then, the PCR products were purified and ligated into pSET4s to construct the deletion vectors pSET4s-potD and pSET4s-arcA, followed by electroporation into *S. suis*-competent cells using a Bio-Rad Gene Pulser Xcell system (Bio-Rad, CA, USA) in THB medium with 0.1 cm cuvettes (2 kV, 1000 Ω, and 25 μF) to obtain the Δ*potD* and Δ*arcA* mutant strains. To construct the complementary strains CΔ*potD* and CΔ*arcA*, the full length of *potD* and *arcA* genes was amplified using primers potD-3-F/potD-3-R and arcA-3-F/arcA-3-R, respectively (Additional file 1). The resulting PCR products were then cloned into the shuttle vector pSET2 to obtain the complemented plasmids pSET2-potD and pSET2-arcA. Finally, the recombinant plasmids were electroporated into Δ*potD *and Δ*arcA *mutant strains to obtain CΔ*potD* and CΔ*arcA*. All mutant strains were verified by PCR amplification and DNA sequencing.

### Bacterial growth curves

SC19, Δ*potD*, and CΔ*potD* in the mid-logarithmic phase were incubated at 37 °C with continuous shaking for 0–12 h. After incubation, the optical density (OD) at 600 nm of the bacterial suspension was measured using a V-1000 spectrophotometer (AOE Instruments, Shanghai, China). Subsequently, bacterial suspension (500 µL) was serially diluted tenfold in sterile phosphate-buffered saline (PBS). From appropriate dilutions, 100 µL aliquots were spread onto THB agar plates. Each dilution was plated in triplicate. After incubation at 37 °C for 24 h, between 30 and 300 colonies were counted on plates.

### Biofilm formation

Bacterial suspension (200 μL) at an OD_600_ of 0.1 was added into a 96-well plate and incubated for 48 h at 37 °C. After incubation, supernatants were removed, and biofilms were washed with PBS three times. Next, biofilms were fixed with 10% methanol (Sigma, St. Louis, USA) for 20 min at room temperature (RT), followed by 1% crystal violet (200 μL/well; BBI, Shanghai, China) staining for 10 min. After the washing steps with PBS, 95% ethanol (Sigma) was added to dissolve the compounds. Finally, biofilm mass was visually assessed and measured by optical absorbance at 570 nm.

### Bacterial infection in vivo

WT C57BL/6 mice (total *N* = 65, each group *n* = 3 or 8) were intraperitoneally injected with 100 μL of PBS (as a blank control) or bacterial suspension containing approximately 2 × 10^8^ colony-forming units (CFU) of SC19, Δ*potD*, or CΔ*potD*. Mice were monitored for 7 days to obtain the survival rate. After 48 h of infection, mice were sacrificed by cervical vertebrae dislocation, and then different tissues, including brain, blood, liver, and lung, were collected to determine bacterial load and tight-junction protein expression. In addition, brains were fixed in 10% formalin (BBI, Shanghai, China) and then embedded in paraffin for hematoxylin and eosin (H&E) staining.

### Evans blue blood–brain barrier permeability assay in vivo

WT C57BL/6 mice (total *N* = 12, each group *n* = 3) were intraperitoneally infected with 100 μL bacterial suspension of SC19, Δ*potD*, and CΔ*potD* (1 × 10^8^ CFU) for 48 h (PBS as a blank control). Then, mice were injected with 200 μL 1% Evans blue (EB; BBI, Shanghai, China) via tail vein, and 2 h after EB administration, they were deeply anesthetized and then transcardially perfused with sterile PBS until the effluent from the right atrium became clear. Subsequently, brains were removed and imaged using a digital camera. Next, brain tissue was homogenized in formamide (BBI, Shanghai, China) on ice. The homogenate was incubated at 37 °C in the dark with shaking for 72 h. After centrifugation at 10 000 *g* for 15 min at 4 °C, the supernatant was collected and filtered, followed by measurement of the absorbance at 632 nm. Finally, EB concentration was calculated on the basis of a standard curve and expressed as micrograms per gram (μg/g) [17].

### Protein expression and purification

The gene sequences of *potD* and *arcA *were amplified from SC19 genome using the primers potD-F/potD-R and arcA-F/arcA-R, which were cloned into pET-28a (for His-tagged fusion) and pGEX-4 T-1 (for GST-tagged fusion), respectively. Then, these recombinant plasmids were transformed into *E. coli* BL21 (DE3) (BBI, Shanghai, China). BL21 (DE3) cells were incubated to mid-log phase with an OD of 0.8 at 600 nm in LB, and then protein expression was induced by the addition of a final concentration of 1 mM isopropyl β-d-1-thiogalactoside (IPTG; BBI, Shanghai, China) for 4 h. Finally, the recombinant His-tagged PotD and GST-tagged ADI proteins were purified using Ni–NTA Elite Beads (Vazyme, Nanjing, China) and BeyoGold^™^ GST-tag Purification Resin (Beyotime, Shanghai, China), respectively. Meanwhile, SC19 cells in the mid-logarithmic phase were harvested by centrifugation and washed with PBS. The pellet was resuspended in PBS and subjected to ultrasonication on ice (300 W output, 3 s pulse on, 5 s pulse off for a total duration of 15 min). The lysate was centrifuged at 12 000 *g* for 30 min at 4 °C. Finally, the supernatant was collected as the total soluble protein extract of *S. suis* SC19. All primers used for target DNA amplification in this study are listed in Additional file 1.

### Mass spectrometry (MS) analysis

To screen for target proteins interacting with PotD, recombinant His-tagged PotD was co-incubated with the total soluble protein extract of SC19 at 4 °C overnight. After incubation, samples were collected and lysed in sodium dodecyl sulfate (SDS) loading buffer (Beyotime). The lysates were subjected to SDS–polyacrylamide gel electrophoresis (PAGE) and Coomassie Brilliant Blue R250 (BBI, Shanghai, China) staining. Subsequently, the stained gels were collected and analyzed by liquid chromatography–mass spectrometry (LC–MS) at Huada Genomics (Beijing, China) to identify specific interacting proteins with PotD.

### GST affinity-isolation assay

Purified GST (10 μg) and GST–ADI (10 μg) were separately mixed with purified His-PotD (20 μg) in 500 μL PBS overnight at 4 °C. The mixtures were then combined with GST-tag Purification Resin (Beyotime) and incubated for 2 h at 4 ℃. The beads were collected by centrifugation at 1000 *g* for 5 min at 4 ℃ and washed with cold PBS. Subsequently, bound proteins on beads were eluted by boiling in SDS loading buffer. For immunoblotting, eluted proteins were separated by SDS–PAGE using a 5% stacking gel and a 10% separating gel and transferred to polyvinylidene difluoride (PVDF) membranes (Vazyme) followed by blocking with 5% nonfat milk in PBST (PBS containing 0.1% Tween-20 [Beyotime]) for 2 h at RT. Then, the membrane was incubated with primary antibodies (Abs) including rabbit anti-GST (1:1000; Proteintech, Wuhan, China, 10000-0-AP) and rabbit anti-His (1:1000; Proteintech, 84814-1-RR) in 5% bovine serum albumin (BSA; BBI, Shanghai, China) in PBST overnight at 4 °C, followed by incubation with peroxidase-conjugated goat anti-rabbit IgG (1:2000; Beyotime, A0208) for 1 h at RT. After washing, proteins were detected using an enhanced chemiluminescence (ECL) detection reagent (Biosharp, Hefei, China) and imaged with a Molecular Imager ChemiDoc XRS + system (Bio-Rad).

### Three dimensional (3D) modeling and protein–protein docking analysis

The structural model of the PotD–ADI interaction complex was performed using GRAMM [18], which employs a rigid-body docking approach. Both the ligand and receptor were treated as rigid structures, which ensured that their conformations remained unchanged throughout the docking process. The algorithm primarily explores potential binding sites on the protein surfaces. The corresponding protein sequences were obtained from UniProtKB [19], followed by the submission to SWISS-MODEL [20] to generate optimal protein structures. The resulting PDB files were subsequently used for the protein–protein docking analysis.

### *S. suis* infection and protein stimulation in cells

For *S. suis* infection, hCMEC/D3, bEnd.3 or pBMECs were infected with SC19, Δ*potD*, CΔ*potD,* Δ*arcA*, or CΔ*arcA* at a multiplicity of infection (MOI) of 10 for 2 h. For protein stimulation, hCMEC/D3 cells were treated with recombinant His-PotD (20 and 40 μg/mL) and GST–ADI (12.5, 25, 50, 100, and 200 μg/mL) for 2 h, respectively. Lipopolysaccharide (LPS) (100 ng/mL) and PBS were included as a positive control and a negative control, respectively. After bacterial infection or protein treatment, cells were lysed, and cell lysates were collected for immunoblotting analysis.

### Western blotting

After cell treatment, cells were lysed in SDS loading buffer. Protein samples were separated by SDS–PAGE using a 5% stacking gel and a 10% or 8% separating gel and subsequently transferred onto a PVDF membrane. The membrane was blocked with 5% (w/v) nonfat dry milk in PBST for 2 h at RT on a shaker. After blocking, the membrane was incubated overnight at 4 °C with primary Abs including rabbit anti-ZO-1 (1:5000; Proteintech, 21773-1-AP) and rabbit anti-Occludin (1:5000; Proteintech, 27260-1-AP). The next day, the membrane was washed with PBST and then incubated with horseradish peroxidase-conjugated goat anti-rabbit IgG (1:2000; Beyotime, A0208) for 1 h at RT. After washing with PBST, the protein bands were visualized using an ECL detection reagent. The chemiluminescent signals were captured using a Molecular Imager ChemiDoc XRS + system. For protein expression quantification, the band intensities were analyzed using ImageJ software and normalized to β-actin.

### Quantitative real-time polymerase chain reaction (RT-qPCR)

Total RNA was isolated from mid-logarithmic phase cultures of SC19, Δ*potD*, and CΔ*potD* strains using the FastPure Total RNA Isolation Kit V2 (Vazyme) and RNA concentration and purity (A_260_/A_280_ ratios between 1.9 and 2.1 and A_260_/A_230_ ratios between 1.9 and 2.2) were determined by Nanodrop. Total RNA (1 μg) was reverse-transcribed into cDNA using the HiScript IV All-in-One Ultra RT SuperMix (Vazyme) according to the manufacturer’s instructions. RT-qPCR was performed on a CFX96 Touch Real-Time PCR Detection System (Bio-Rad) using ChamQ Blue Universal SYBR qPCR Master Mix (Vazyme). The 16S rRNA gene was used as the reference gene for normalization. The 16S rRNA gene exhibited a standard deviation (SD) < 1 and was significantly correlated with the BestKeeper index (*p* < 0.05) across all datasets, in both pooled and group-specific analysis. These results confirm its suitability as a reference gene under the present experimental conditions. The amplification efficiencies for all primers were between 90% and 110%, and the correlation coefficients (*R*^2^) for the standard curves were greater than 0.98. RT-qPCR was performed as an initial denaturation at 95 °C for 30 s, followed by 40 cycles of 95 °C for 10 s and 60 °C for 30 s. Gene expression in the Δ*potD* and CΔ*potD* strains was calculated relative to the wild-type SC19 control using the 2^−ΔΔCT^ method [21]. The sequences of all primers and their corresponding GenBank accession numbers, amplicon sizes, and amplification efficiencies are listed in Additional file 1.

### Statistical analysis

Data are represented as mean ± standard error of the mean (SEM) of three independent experiments for each group (*n* = 3 or 8). One-way analysis of variance (ANOVA) was used to analyze the statistical differences for comparisons among different groups. *P*-values ≤ 0.05 were considered statistically significant. *P*-values for all statistical comparisons are shown in the figures.

## Results

### *potD* deletion reduces biofilm formation and pathogenicity of *Streptococcus suis*

**Figure 1:**
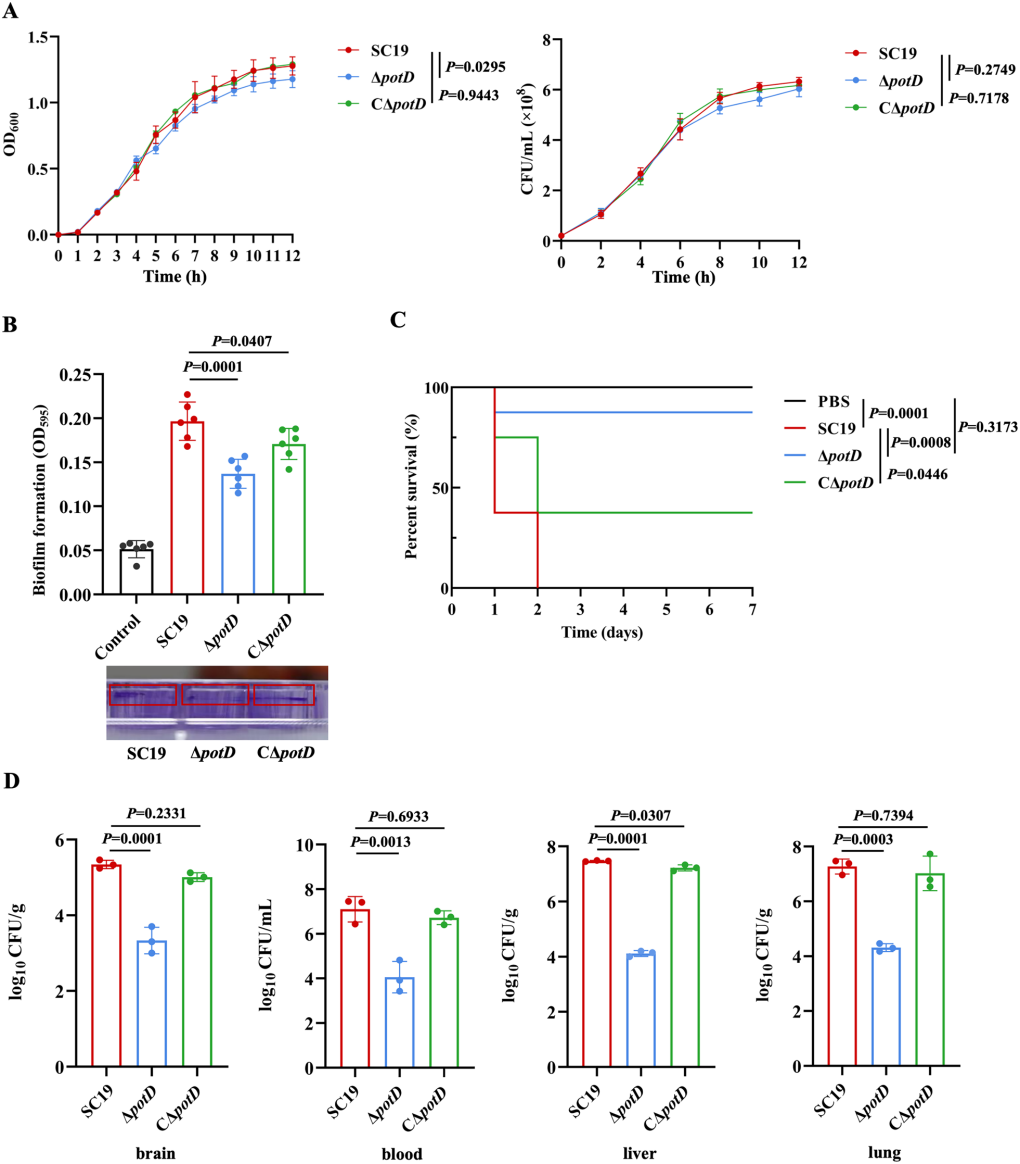
***potD*** **deletion reduces biofilm formation and pathogenicity of** ***Streptococcus suis***. **A** Growth curves of SC19, Δ*potD*, and CΔ*potD* measured by OD_600_ (left panel) and CFU/mL (right panel) at 37 °C. **B** Crystal violet staining of biofilms of SC19, Δ*potD*, and CΔ*potD*.** C** Survival curves of C57BL/6 mice infected with PBS, SC19, Δ*potD*, and CΔ*potD* (total *N* = 32, each group *n* = 8). **D** Bacterial load in the brain, blood, liver, and lung of mice infected with SC19, Δ*potD*, and CΔ*potD* for 48 h (total *N* = 9, each group *n* = 3). **A** and **B** are representative of three independent experiments with triplicate samples per group. **C** and **D** are representative of three independent experiments with three or eight mice per group.

To investigate the effect of PotD on *S. suis* pathogenicity, we constructed *potD* knockout mutant strain (Δ*potD*) and complementary mutant strain (CΔ*potD*) under the background of SC19 strain (Additional files 2A and B). The results showed that *potD* deletion slightly affected *S. suis* growth, which was consistent with the viable bacterial counts (Figure 1A). Compared with WT SC19, *potD* deletion significantly reduced biofilm formation, whereas CΔ*potD* rescued such reduction (Figure 1B). To further explore the effect of PotD on *S. suis* pathogenicity in vivo, mice were intraperitoneally infected with SC19, Δ*potD*, and CΔ*potD*. The results showed that Δ*potD* and CΔ*potD* exhibited higher survival rates than SC19 (Figure 1C). Similarly, bacterial loads in the brain, blood, liver, and lung were significantly lower in mice infected with Δ*potD* than in those infected with SC19. In contrast, bacterial loads in mice infected with CΔ*potD* were similar to those in the SC19-infected group. (Figure 1D). These results suggest that PotD is involved in the *S. suis* pathogenicity in murine model.

### potD deletion reduces *Streptococcus suis*-induced blood–brain barrier disruption

**Figure 2:**
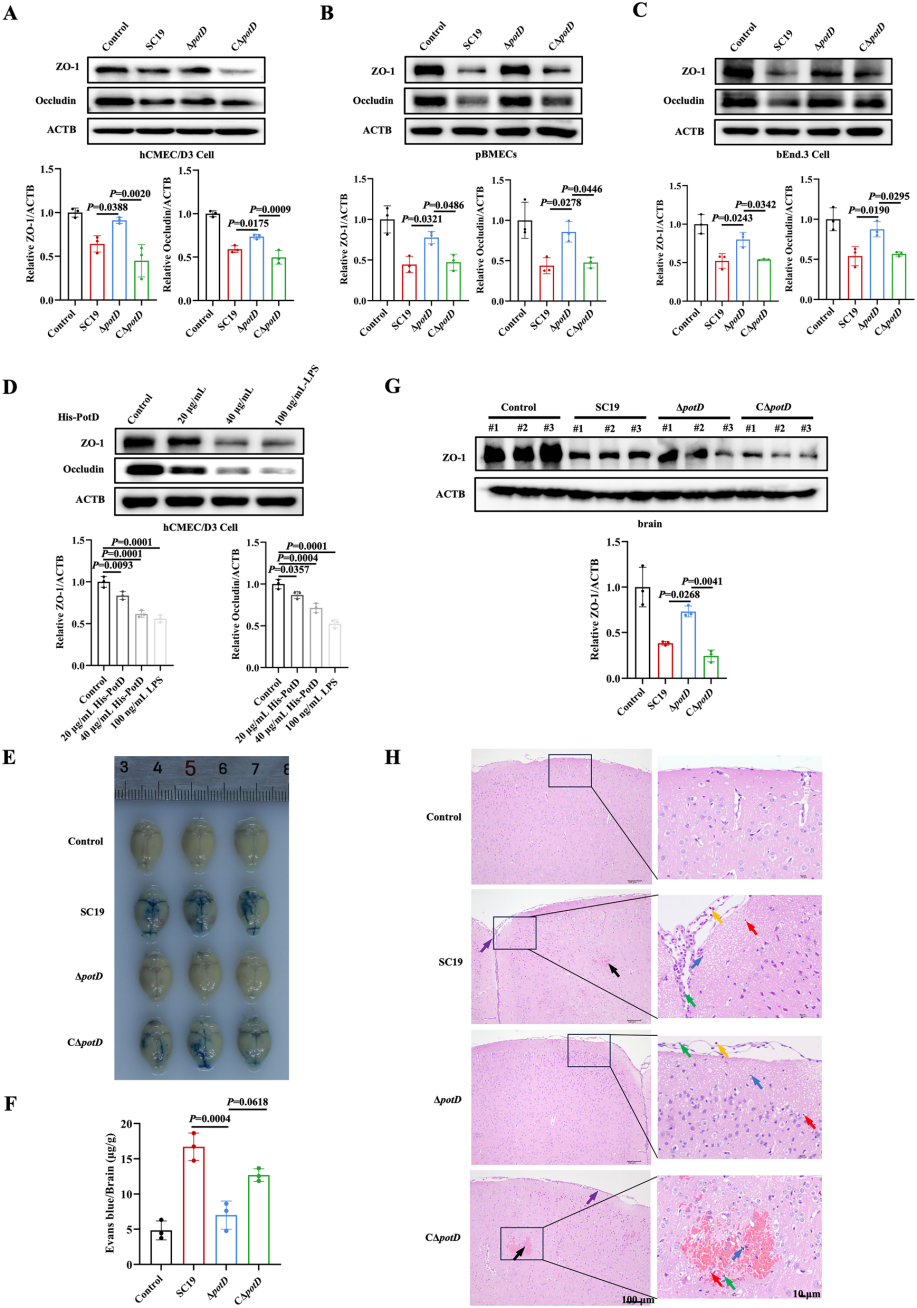
***potD*** **deletion reduces** ***Streptococcus suis*-induced blood–brain barrier disruption.** **A–C** Immunoblot analysis of ZO-1 and occludin expression in hCMEC/D3, pBMECs, and bEnd.3 cells infected with SC19, Δ*potD*, and CΔ*potD*. **D** Immunoblot analysis of ZO-1 and occludin expression in hCMEC/D3 cells stimulated with recombinant His-PotD and LPS for 2 h. C57BL/6 mice were intraperitoneally inoculated with PBS, SC19, Δ*potD*, and CΔ*potD* for 48 h. After infection, brains were collected for the indicated assays. **E** Representative images of EB diffusion in the mouse brain (total *N* = 12, each group *n* = 3). **F** Quantification of EB penetration in the mouse brain. **G** Immunoblot analysis of ZO-1 in brain (total *N* = 12, each group *n* = 3). **H** Representative images of histopathological changes in the brain via H&E staining (total *N* = 12, each group *n* = 3). Pathological features include hemorrhage (black arrows), inflammatory cell infiltration (purple arrows), necrotic cellular debris (red arrows), lymphocytes (yellow arrows), neutrophils (green arrows), and activated glial cells (blue arrows). All data are representative of three independent experiments with triplicate samples per group.

BBB is a defensive structure in the central nervous system, which is composed of brain microvascular endothelial cells (BMECs), glial cells, and pericytes, of which BMECs serve as the first line of defense in the BBB against microbial infection. The functional integrity of BMECs is maintained by tight junctions (TJs), including occludin, claudin, junctional adhesion molecules (JAMs), and zonula occludens-1 (ZO-1) [22]. Since BBB invasion is a key pathogenicity mechanism of *S. suis*, we further investigated the effect of PotD on *S. suis*-induced BBB disruption in vitro and in vivo. The results showed that SC19 significantly downregulated the expression of ZO-1 and occludin in hCMEC/D3, pBMECs, and bEnd.3 cells, whereas Δ*potD* reversed this downregulation (Figures 2A–C). However, CΔ*potD* showed similar effect with SC19 on the downregulation of ZO-1 and occludin. Furthermore, recombinant His-tagged PotD (His-PotD) downregulated the expression of ZO-1 and occludin in hCMEC/D3 (Figure 2D). In the *S. suis-*infected mice model, EB staining assay revealed that SC19 and CΔ*potD* increased BBB permeability with EB dye diffusion in the brain, whereas Δ*potD* did not affect BBB permeability (Figures 2E, F). Consistent with BBB permeability in the brain, *potD* knockout rescued *S. suis*-induced downregulation of ZO-1 (Figure 2G). During SC19 infection, pathological alterations, including hemorrhages, neuronal degeneration, necrosis, increased inflammatory cell infiltration, and glial cell proliferation were observed (Figure 2H). The pathological damage caused by Δ*potD* is similar to that caused by SC19, but there is no bleeding symptom (Figure 2H). These results demonstrate that PotD mediates *S. suis*-induced BBB disruption.

### PotD interacts with ADI and mediates *arcA* transcription

**Figure 3:**
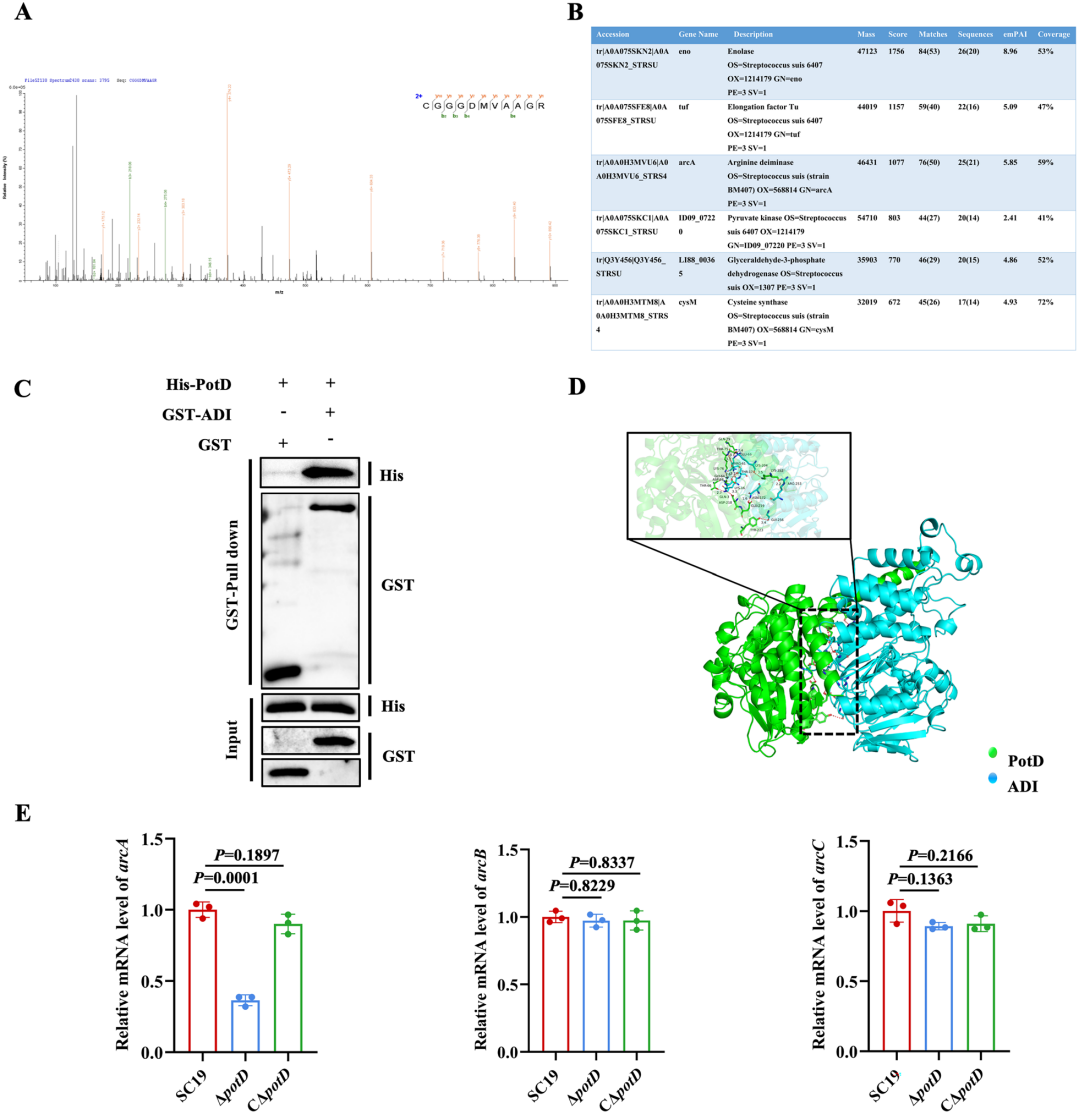
***PotD*** **interacts with ADI and mediates** ***arcA*** **transcription.** **A, B** Mass spectrometry analysis of PotD protein** (A)** and its potential interacting proteins** (B)**. **C** PotD–ADI interaction by GST pulldown assay. **D** Structural model of PotD–ADI interaction. **E** mRNA expression of *arcA*, *arcB*, and *arcC* in SC19, Δ*potD*, and CΔ*potD* strains. **C** and **E** are representative of three independent experiments with triplicate samples per group.

To further investigate the underlying mechanism by which PotD mediates BBB, we constructed recombinant His-PotD protein and co-incubated it with the total soluble protein extract of *S. suis* SC19 to screen the interaction proteins with PotD through mass spectrometry (MS) analysis. We firstly prepared the total soluble protein extract of *S. suis* SC19 by ultrasonic disruption. Then, the recombinant His-PotD protein was purified and its purity verified by SDS–PAGE (Additional file 2C), followed by incubation with soluble protein extract of SC19. The mixture obtained after incubation was separated by SDS–PAGE (Additional file 2E) and subsequently analyzed by MS to identify potential interacting proteins. MS revealed several potential PotD-interacting proteins, including enolase, elongation factor Tu, arginine deiminase (ADI), pyruvate kinase, glyceraldehyde-3-phosphate dehydrogenase, and cysteine synthase (Figures 3A, B). Owing to the lack of de novo synthesis pathway of polyamines, the biosynthesis of endogenous polyamines relies on intermediates from arginine metabolism, in which ADI serves as a key catalytic enzyme, suggesting that ADI plays a critical role in polyamine synthesis [23]. In addition, streptococcal ADI disrupts the actin cytoskeleton structure of endothelial cells [24]. Therefore, we further focused on the identification of the PotD–ADI interaction. To validate this interaction, we firstly expressed and purified GST–ADI protein (Additional file 2D) for the use of pulldown assay. The results of GST pulldown assay showed that His-tagged PotD was immunoprecipitated by GST-tagged ADI, indicating the interaction between PotD and ADI (Figure 3C). To further characterize this binding, molecular docking was performed. Structural model analysis predicted that PotD and ADI interact via surface complementarity with a binding free energy of −8.8 kcal/mol, suggesting the stable interaction (Figure 3D). In addition, we found that PotD deficiency significantly reduced mRNA expression of *arcA* encoding ADI (Figure 3E). In contrast to its effect on *arcA*, PotD deficiency did not alter the transcriptional level of *arcB* or *arcC* (Figure 3E), demonstrating that PotD specifically regulates *arcA*. These results demonstrate that PotD mediates arginine metabolism via interaction with ADI.

### PotD-mediated *arcA* regulates *Streptococcus suis*-induced blood–brain barrier disruption

**Figure 4:**
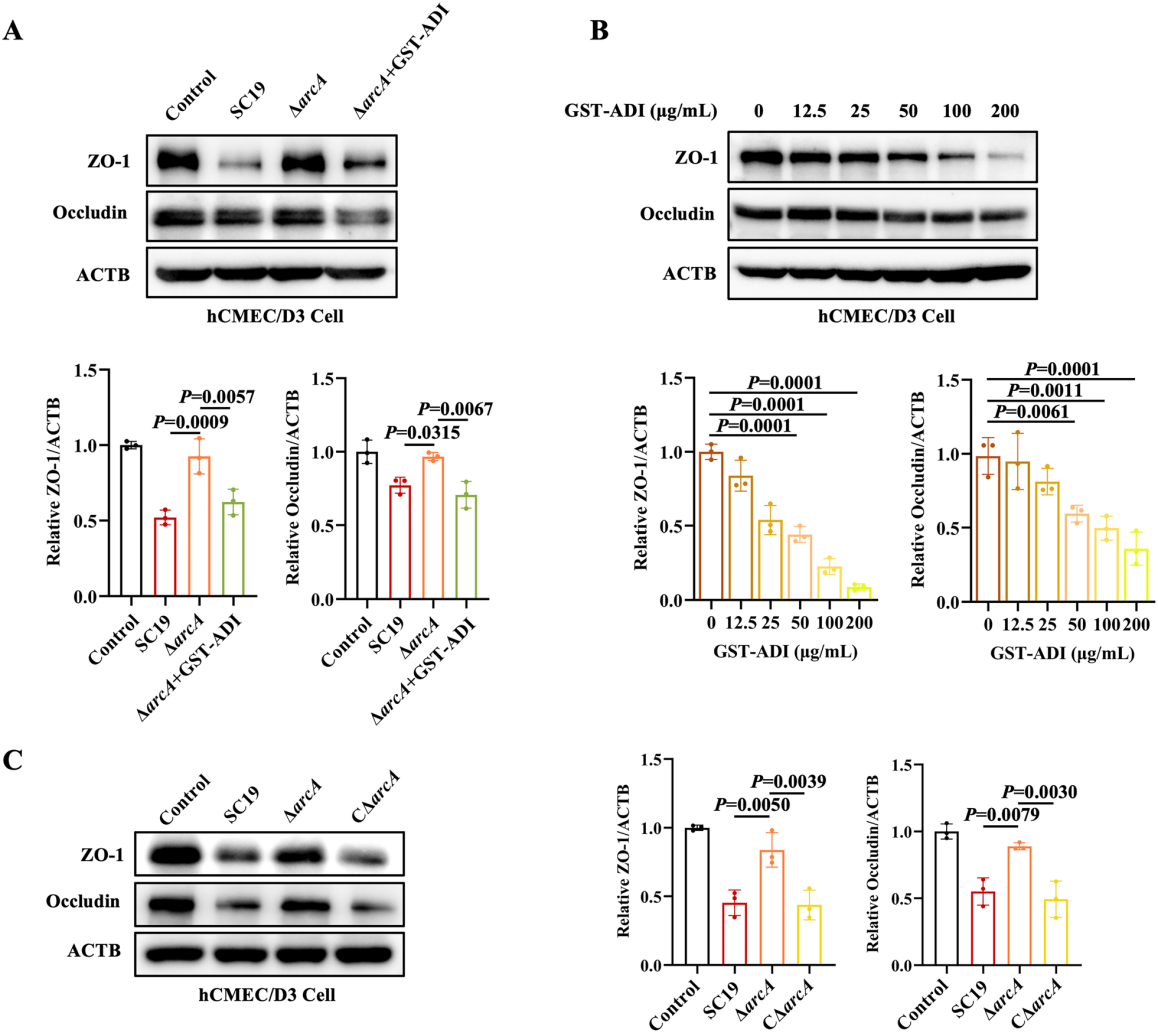
**PotD-mediated** ***arcA*** **regulates** ***Streptococcus suis*-induced blood–brain barrier disruption.** **A** Immunoblot analysis of ZO-1 and occludin in hCMEC/D3 cells infected with SC19, Δ*arcA*, and Δ*arcA* + GST–ADI. **B** Immunoblot analysis of ZO-1 and occludin in hCMEC/D3 cells infected with GST–ADI at the indicated concentrations. **C** Immunoblot analysis of ZO-1 and occludin expression in hCMEC/D3 cells infected with SC19, Δ*arcA*, and CΔ*arcA*. All data are representative of three independent experiments with triplicate samples per group.

To further investigate the role of *arcA* in *S. suis*-induced BBB disruption, we constructed *arcA* knockout mutant strain (Δ*arcA*) under the background of SC19. The results showed that SC19 significantly reduced the expression of ZO-1 and occludin, whereas Δ*arcA* rescued this reduction (Figure 4A). Moreover, exogenous supplementation of ADI significantly reduced the expression of ZO-1 and occludin (Figure 4A). Notably, recombinant ADI downregulated the expression of ZO-1 and occludin in a concentration-dependent manner (Figure 4B). Consistent with this finding, SC19 and CΔ*arcA* strains downregulated the expression of ZO-1 and occludin, while Δ*arcA* reversed this downregulation (Figure 4C). These results indicate that ADI is involved in *S. suis*-induced BBB disruption.

## Discussion

Our study reveals the critical role of PotD in *S. suis* pathogenesis by constructing Δ*potD* and CΔ*potD*, especially in the BBB disruption. Our findings are consistent with the virulence-related role of PotD in other bacteria, such as *Actinobacillus pleuropneumoniae* [25], *S. pneumoniae* [7], *Glaesserella parasuis* (*G. parasuis*) [26], and *Legionella pneumophila* (*L. pneumophila*) [10]. The mutant Δ*potD* displays attenuated pathogenesis phenotypes in biofilm formation [7], oxidative tolerance [25], Na^+^ tolerance or filament formation [10]. Our study showed that PotD deletion attenuated *S. suis*-induced blood–brain barrier disruption and biofilm formation, indicating that PotD mediates bacterial virulence by different pathogenesis phenotypes. In addition to involvement in bacterial pathogenesis, PotD has been reported as a potential vaccine candidate. Immunization with recombinant PotD protects mice against *S. pneumoniae* [27] and *G. parasuis* [28]. PotD of *G. parasuis* activates TLR4 to induce JNK/MAPK/NF-κB-mediated inflammatory response in murine macrophages [28]. However, our study showed the destructive role of PotD in brain endothelial cells. Whether PotD protects the host against *S. suis* infection needs to be further studied.

Polyamines mediate various biological processes in bacteria, such as biofilm formation, toxin activity, host cell apoptosis, and oxidative and acid tolerance [29]. Bacterial polyamine pools are controlled by endogenous biosynthesis and exogenous transport. Unlike the de novo synthesis pathway of polyamine present in *S. pneumoniae* [30], exogenous polyamine transport is critical for *S. suis* pathogenicity owing to the lack of de novo synthesis pathway [11]. PotABCD in *S. suis* has been functionally characterized for polyamine uptake using Δ*potA*, wherein *potABCD* genes are co-transcribed by *murB* to regulate peptidoglycan (PG) synthesis, and PotD is confirmed to bind to the protein of polyamines [11], suggesting that PotD serves as a primary receptor for polyamine transport in *S. suis*. Theoretically, PotD knockout could impair the bacterial growth, as PotD plays a critical role in the uptake of polyamines. However, under the conditions of our study, no significant growth defect was observed, indicating that another potential compensatory mechanism might exist. Intracellular polyamines mediate transcription and translation through RNA polymerase [31–33]. In *E. coli*, the addition of polyamines increases the level of RNA polymerase RpoS, resulting in *gadE* expression for the glutamate-dependent acid resistance system [32]. Impaired polyamine synthesis results in reduced expression of transport genes in *S. pneumoniae* [34]. In *S. suis*, the addition of polyamines increases the expression of genes encoding transport and PG synthesis, whereas impaired polyamine transport by the deletion of *potA* disrupts expression of genes involved in PG synthesis [11]. It has been reported that polyamines regulate the expression of proteins involved in pneumococcal virulence [35]. Our study showed that PotD interacted with ADI and mediated the expression of *arcA* encoding ADI protein involved in *S. suis*-induced BBB disruption.

Our study also showed that *arcA* encoding ADI contributed to *S. suis* virulence via BBB disruption, which is consistent with attenuated virulence in ΔADS with increased arginine and antimicrobial nitric oxide (NO) production in macrophages, decreased intracellular survival, and decreased bacterial virulence in a mouse infection model [36]. In addition, the transcriptional regulator XtrSs in *S. suis* directly binds to ArgR and negatively regulates *arcA* expression. XtrSs knockout enhances bacterial acid resistance by upregulating *arcA* expression [36]. Although our study lacks the investigation of the effect of *arcA* on bacterial acid resistance, our study showed that PotD bound to ADI and Δ*potD* downregulated *arcA* expression, leading to attenuated BBB disruption. These studies demonstrate that ADS is regulated by different factors, and whether PotD regulates *arcA* through ArgR and XtrSs needs to be further studied.

In conclusion, this study reveals a key virulence factor, PotD, of *S. suis* and elucidates a novel interplay between PotD and ADI in regulating *S. suis*-induced BBB disruption. These findings provide new insight into the pathogenic mechanism of *S. suis* and potential therapeutic targets to control *S. suis* infection.

## Supplementary Information

**Additional file** **1** **The primers used in this study.**

**Additional file**  **2**. **Results of PCR validation of Δ*potD*** **and CΔ*potD*** **and purification of recombinant PotD protein and ADI protein.** **A, B** PCR verification of Δ*potD* mutant **(A)** and complementary mutant strain CΔ*potD (B)*. **C** Purification of His-tagged PotD protein. Lane M: Protein marker (180 kDa); Lane 1: Unpurified cell lysate; Lane 2: Proteins removed with 10 mM imidazole buffer; Lane 3: Proteins removed with 20 mM imidazole buffer; Lane 4: His-PotD protein eluted with 250 mM imidazole buffer. **D** Purification of GST-tagged ADI protein. Lane M: Protein marker (250 kDa); Lanes 1–8: GST-fused protein eluted with 10 mM reduced glutathione in elution buffer. The target proteins are denoted by red boxes. **E** SDS–PAGE analysis of total soluble protein extract of SC19 and purified His-PotD. Lane M: Protein marker (180 kDa); Lane 1: Total soluble protein extract of SC19; Lane 2: Purified His-PotD; Lane 3: Mixture of total soluble protein extract of SC19 and His-PotD. The excised gel band (Lane 3) was subjected to mass spectrometry analysis.

## Data Availability

The datasets used and/or analyzed during the current study are available from the corresponding author on reasonable request.

## References

[1] Haas B, Grenier D (2018) Understanding the virulence of *Streptococcus suis*: a veterinary, medical, and economic challenge. Med Mal Infect 48:159–166 29122409 10.1016/j.medmal.2017.10.001

[2] van Samkar A, Brouwer MC, Schultsz C, van der Ende A, van de Beek D (2015) *Streptococcus suis* meningitis: a systematic review and meta-analysis. PLoS Negl Trop Dis 9:e0004191 26505485 10.1371/journal.pntd.0004191 PMC4624688

[3] Segura M, Fittipaldi N, Calzas C, Gottschalk M (2017) Critical *Streptococcus suis* virulence factors: are they all really critical? Trends Microbiol 25:585–599 28274524 10.1016/j.tim.2017.02.005

[4] Tram G, Jennings MP, Blackall PJ, Atack JM (2021) *Streptococcus suis* pathogenesis-A diverse array of virulence factors for a zoonotic lifestyle. Adv Microb Physiol 78:217–257 34147186 10.1016/bs.ampbs.2020.12.002

[5] Zahedi K, Barone S, Soleimani M (2022) Polyamines and their metabolism: from the maintenance of physiological homeostasis to the mediation of disease. Med Sci 10:38 10.3390/medsci10030038 PMC9326668 35893120

[6] Qiao Z, Do PH, Yeo JY, Ero R, Li Z, Zhan L, Basak S, Gao YG (2024) Structural insights into polyamine spermidine uptake by the ABC transporter PotD-PotABC. Sci Adv 10:eado8107 39303029 10.1126/sciadv.ado8107 PMC11414716

[7] Pipkins HR, Bradshaw JL, Keller LE, Swiatlo E, McDaniel LS (2017) Polyamine transporter potABCD is required for virulence of encapsulated but not nonencapsulated *Streptococcus pneumoniae*. PLoS One 12:e0179159 28586394 10.1371/journal.pone.0179159 PMC5460881

[8] Shah P, Romero DG, Swiatlo E (2008) Role of polyamine transport in *Streptococcus pneumoniae* response to physiological stress and murine septicemia. Microb Pathog 45:167–172 18572376 10.1016/j.micpath.2008.05.001

[9] Zhang X, Zhang Y, Liu J, Liu H (2013) PotD protein stimulates biofilm formation by *Escherichia coli*. Biotechnol Lett 35:1099–1106 23539287 10.1007/s10529-013-1184-8

[10] Nasrallah GK, Abdelhady H, Tompkins NP, Carson KR, Garduño RA (2014) Deletion of potD, encoding a putative spermidine-binding protein, results in a complex phenotype in *Legionella pneumophila*. Int J Med Microbiol 304:703–716 24928741 10.1016/j.ijmm.2014.05.004

[11] Liu W, Tan M, Zhang C, Xu Z, Li L, Zhou R (2018) Functional characterization of *murB-potABCD* operon for polyamine uptake and peptidoglycan synthesis in *Streptococcus suis*. Microbiol Res 207:177–187 29458852 10.1016/j.micres.2017.11.008

[12] Xiong L, Teng JL, Botelho MG, Lo RC, Lau SK, Woo PC (2016) Arginine metabolism in bacterial pathogenesis and cancer therapy. Int J Mol Sci 17:363 26978353 10.3390/ijms17030363 PMC4813224

[13] Fulde M, Willenborg J, de Greeff A, Benga L, Smith HE, Valentin-Weigand P, Goethe R (2011) ArgR is an essential local transcriptional regulator of the arcABC operon in *Streptococcus suis* and is crucial for biological fitness in an acidic environment. Microbiology (Reading) 157:572–582 20947575 10.1099/mic.0.043067-0

[14] Li W, Liu L, Qiu D, Chen H, Zhou R (2010) Identification of *Streptococcus suis* serotype 2 genes preferentially expressed in the natural host. Int J Med Microbiol 300:482–488 20554247 10.1016/j.ijmm.2010.04.018

[15] Takamatsu D, Osaki M, Sekizaki T (2001) Thermosensitive suicide vectors for gene replacement in *Streptococcus suis*. Plasmid 46:140–148 11591139 10.1006/plas.2001.1532

[16] Xu B, Yang X, Zhang P, Ma Z, Lin H, Fan H (2016) The arginine deiminase system facilitates environmental adaptability of *Streptococcus equi* ssp. *zooepidemicus* through pH adjustment. Res Microbiol 167:403–412 27068185 10.1016/j.resmic.2016.03.005

[17] Jiang X, Li F, Mei J, Wu T, Zhu J, Li Z, Wu Z, Jiang H, Li N, Lei L (2024) Brain immune cell infiltration and serum metabolomic characteristics reveal that lauric acid promotes immune cell infiltration in brain and *Streptococcus suis* meningitis in mice. Mol Neurobiol 61:9302–9319 38625620 10.1007/s12035-024-04144-1

[18] GRAMM: protein docking web service. http://gramm.compbio.ku.edu/. Accessed 25 May 2025

[19] UniProtKB. https://www.uniprot.org. Accessed 25 May 2025

[20] SWISS-MODEL. https://swissmodel.expasy.org. Accessed 25 May 2025

[21] Livak KJ, Schmittgen TD (2001) Analysis of relative gene expression data using real-time quantitative PCR and the 2(-Delta Delta C(T)) method. Methods 25:402–408 11846609 10.1006/meth.2001.1262

[22] Heinemann U, Schuetz A (2019) Structural features of tight-junction proteins. Int J Mol Sci 20:6020 31795346 10.3390/ijms20236020 PMC6928914

[23] Fung TS, Ryu KW, Thompson CB (2025) Arginine: at the crossroads of nitrogen metabolism. EMBO J 44:1275–1293 39920310 10.1038/s44318-025-00379-3 PMC11876448

[24] Mammedova JT, Sokolov AV, Burova LA, Karaseva AB, Grudinina NA, Gorbunov NP, Malashicheva AB, Semenova DS, Kiseleva EP, Starikova EA (2023) Streptococcal arginine deiminase regulates endothelial inflammation, mTOR pathway and autophagy. Immunobiology 228:152344 36746072 10.1016/j.imbio.2023.152344

[25] Zhu Z, Zhao Q, Zhao Y, Zhang F, Wen X, Huang X, Wen Y, Wu R, Yan Q, Huang Y, Ma X, Han X, Cao S (2017) Polyamine-binding protein PotD2 is required for stress tolerance and virulence in *Actinobacillus pleuropneumoniae*. Antonie Van Leeuwenhoek 110:1647–1657 28733844 10.1007/s10482-017-0914-7

[26] Dai K, Yang Z, Ma X, Chang YF, Cao S, Zhao Q, Huang X, Wu R, Huang Y, Xia J, Yan Q, Han X, Ma X, Wen X, Wen Y (2021) Deletion of polyamine transport protein PotD exacerbates virulence in *Glaesserella* (*Haemophilus*) *parasuis* in the form of non-biofilm-generated bacteria in a murine acute infection model. Virulence 12:520–546 33525975 10.1080/21505594.2021.1878673 PMC7872090

[27] Shah P, Briles DE, King J, Hale Y, Swiatlo E (2009) Mucosal immunization with polyamine transport protein D (PotD) protects mice against nasopharyngeal colonization with *Streptococcus pneumoniae*. Exp Biol Med 234:403–409 10.3181/0809-RM-269 19176871

[28] Dai K, Ma X, Yang Z, Chang YF, Cao S, Zhao Q, Huang X, Wu R, Huang Y, Yan Q, Han X, Ma X, Wen X, Wen Y (2019) Polyamine transport protein PotD protects mice against *Haemophilus parasuis* and elevates the secretion of pro-inflammatory cytokines of macrophage via JNK-MAPK and NF-κB signal pathways through TLR4. Vaccines 7:216 10.3390/vaccines7040216 PMC6963478 31847381

[29] Shah P, Swiatlo E (2008) A multifaceted role for polyamines in bacterial pathogens. Mol Microbiol 68:4–16 18405343 10.1111/j.1365-2958.2008.06126.x

[30] Potter AJ, Paton JC (2014) Spermidine biosynthesis and transport modulate pneumococcal autolysis. J Bacteriol 196:3556–3561 25092031 10.1128/JB.01981-14 PMC4187697

[31] Miller-Fleming L, Olin-Sandoval V, Campbell K, Ralser M (2015) Remaining mysteries of molecular biology: the role of polyamines in the cell. J Mol Biol 427:3389–3406 26156863 10.1016/j.jmb.2015.06.020

[32] Chattopadhyay MK, Keembiyehetty CN, Chen W, Tabor H (2015) Polyamines stimulate the level of the σ38 subunit (RpoS) of *Escherichia coli* RNA polymerase, resulting in the induction of the glutamate decarboxylase-dependent acid response system via the gadE regulon. J Biol Chem 290:17809–17821 26025365 10.1074/jbc.M115.655688 PMC4505032

[33] Igarashi K, Kashiwagi K (2018) Effects of polyamines on protein synthesis and growth of *Escherichia coli*. J Biol Chem 293:18702–18709 30108177 10.1074/jbc.TM118.003465 PMC6290148

[34] Nakamya MF, Ayoola MB, Park S, Shack LA, Swiatlo E, Nanduri B (2018) The role of cadaverine synthesis on pneumococcal capsule and protein expression. Med Sci (Basel) 6:8 10.3390/medsci6010008 PMC5872165 29351189

[35] Shah P, Nanduri B, Swiatlo E, Ma Y, Pendarvis K (2011) Polyamine biosynthesis and transport mechanisms are crucial for fitness and pathogenesis of *Streptococcus pneumoniae*. Microbiology 157:504–515 20966092 10.1099/mic.0.042564-0

[36] Zhang Y, Liang S, Zhang S, Bai Q, Dai L, Wang J, Yao H, Zhang W, Liu G (2024) Streptococcal arginine deiminase system defences macrophage bactericidal effect mediated by XRE family protein XtrSs. Virulence 15:2306719 38251714 10.1080/21505594.2024.2306719 PMC10841013

