# Supplementary material for: PotD contributes to Streptococcus suis-induced blood–brain barrier disruption by regulating arcA transcription
Source: Vet Res. 2025 Dec 1;57:5. doi: 10.1186/s13567-025-01676-9 (PMC12777399; doi:10.1186/s13567-025-01676-9)
Supplement: Supplementary file 2 — Additional file 2. Results of PCR validation of ΔpotD and CΔpotD and purification of recombinant PotD protein and ADI protein. A, B PCR verification of ΔpotD mutant (A) and complementary mutant strain CΔpotD (B). C Purification of His-tagged PotD protein. Lane M: Protein marker (180 kDa); Lane 1: Unpurified cell lysate; Lane 2: Proteins removed with 10 mM imidazole buffer; Lane 3: Proteins removed with 20 mM imidazole buffer; Lane 4: His-PotD protein eluted with 250 mM imidazole buffer. D Purification of GST-tagged ADI protein. Lane M: Protein marker (250 kDa); Lanes 1–8: GST-fused protein eluted with 10 mM reduced glutathione in elution buffer. The target proteins are denoted by red boxes. E SDS–PAGE analysis of total soluble protein extract of SC19 and purified His-PotD. Lane M: Protein marker (180 kDa); Lane 1: Total soluble protein extract of SC19; Lane 2: Purified His-PotD; Lane 3: Mixture of total soluble protein extract of SC19 and His-PotD. The excised gel band (Lane 3) was subjected to mass spectrometry analysis. [file 13567_2025_1676_MOESM2_ESM.docx]

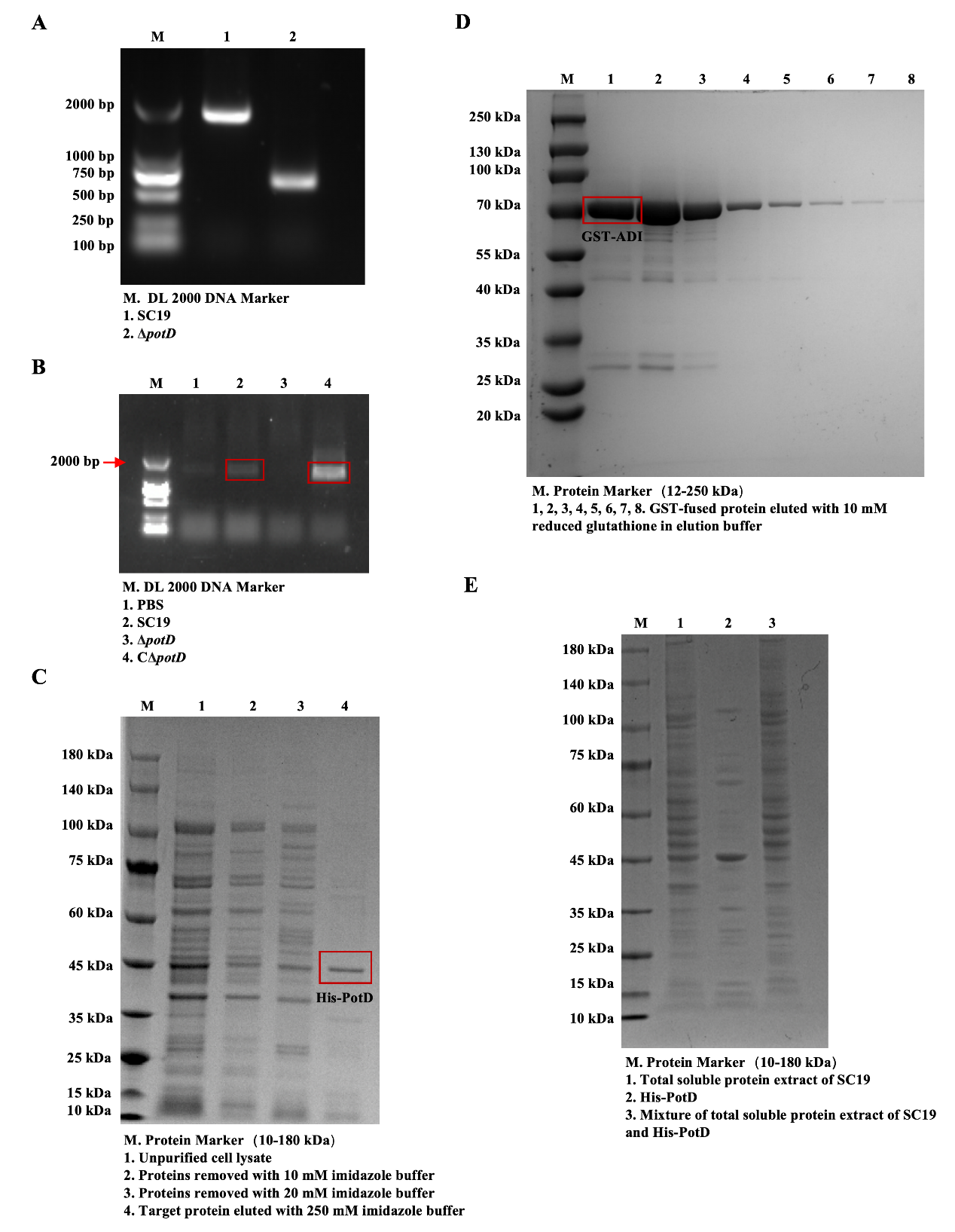
**Additional file 1. Results of PCR validation of Δ*****potD* and CΔ*potD* and purification of recombinant PotD protein and ADI protein.** **A, B** PCR verification of Δ*potD* mutant **(A)** and complementary mutant strain CΔ*potD* **(B)**. **C** Purification of His-tagged PotD protein. Lane M: Protein marker (180 kDa); Lane 1: Unpurified cell lysate; Lane 2: Proteins removed with 10 mM imidazole buffer; Lane 3: Proteins removed with 20 mM imidazole buffer; Lane 4: His-PotD protein eluted with 250 mM imidazole buffer. **D** Purification of GST-tagged ADI protein. Lane M: Protein marker (250 kDa); Lanes 1-8: GST-fused protein eluted with 10 mM reduced glutathione in elution buffer. The target proteins are denoted by red boxes. **E** SDS-PAGE analysis of total soluble protein extract of SC19 and purified His-PotD. Lane M: Protein marker (180 kDa); Lane 1: Total soluble protein extract of SC19; Lane 2: Purified His-PotD; Lane 3: Mixture of total soluble protein extract of SC19 and His-PotD. The excised gel band (Lane 3) was subjected to mass spectrometry analysis.
